# The subapical labial sensory organ of spotted lanternfly *Lycorma delicatula*

**DOI:** 10.1098/rsob.230438

**Published:** 2024-03-27

**Authors:** Hany K. M. Dweck, Claire E. Rutledge

**Affiliations:** Department of Entomology, The Connecticut Agricultural Experiment Station, New Haven, CT 06511, USA

**Keywords:** spotted lanternfly, labium, placoid sensilla, wind, odorants

## Abstract

Deciphering how spotted lanternfly (SLF), an invasive polyphagous planthopper in North America, engages with its environment is a pressing issue with fundamental biological significance and economic importance. This interaction primarily depends on olfaction. However, the cellular basis of olfaction in SLF remains elusive. Here we investigate the neuronal and functional organization of the subapical labial sensory organ using scanning electron microscopy and electrophysiological recordings. This organ is believed to supply planthoppers with crucial sensory information that influences their subsequent feeding behaviour. We find in SLF that this organ comprises two identical placoid sensilla, each housing two distinct neurons. The A neuron displays a remarkable sensitivity to changes in airflow speed. Importantly, the same neuron also exhibits robust excitatory responses exclusively to three aldehydes out of a diverse pool of 85 tested odorants and inhibitory responses to 62 other odorants. By contrast, the B neuron solely serves as an olfactory detector, showing strong excitatory responses to 17 odorants and inhibitory responses to only three. The results provide a potential cellular basis for the behavioural responses of SLF to its ecologically relevant stimuli. Our study also identifies new odorants that may be useful for managing this serious pest.

## Graphical abstract

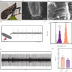

## 1. Introduction

Spotted lanternfly (SLF), *Lycorma delicatula* White (Hemiptera: Fulgoridae), native to China, Japan, Vietnam, has recently invaded North America. The initial detection of SLF in the United States occurred in Berks County, Pennsylvania in late 2014 [1].

Both adults and nymphs (four instars) of this insect are sap feeders with the potential to cause severe damage to agricultural crops, including hop yards, nurseries, orchards and vineyards [1–4]. The tree-of-heaven (*Ailanthus altissima*), which serves as the preferred host for SLF [1,3–6], is a highly invasive species and is abundant along highways, in urban areas, and along the borders of agricultural and industrial zones. These areas provide ideal conditions for the establishment of SLF.

In addition to its feeding behaviour, which induces sap leakage from trees, SLF also produces a substantial amount of liquid excrement known as honeydew [1,4,7]. Honeydew primarily consists of water, sugar, and amino acids [8,9]. When combined with the sap oozing from infested trees, honeydew facilitates the growth of sooty mould, which interferes with photosynthesis [2,10]. Furthermore, the honeydew can rain down on various surfaces, vehicles, and individuals in infested areas [4], creating unpleasant and unsanitary situations. Moreover, the sweet nature of honeydew tends to attract ants, stinging bees, wasps, and other sugar-loving insects [4,11], exacerbating the overall problem. In many cases, trees affected by SLF emit a distinct fermented smell [4], adding to the complexity of the situation.

The presence of SLF can also have national and international implications, as other states or countries may refuse to accept agricultural exports from US states with known SLF populations if they suspect contamination by this pest. The gravity of the situation calls for immediate action to address the challenges posed by SLF. By understanding how SLF interacts with its environment, we can develop effective strategies to manage and mitigate its spread.

Like many insects, several aspects of the interaction of SLF with its environment are primarily mediated by olfactory cues [6,7,12,13]. These include finding host plants, selecting suitable feeding sites, aggregating, mating, finding ideal spots for depositing egg masses, and avoiding predators. Thus, identifying the olfactory cues that drive these behaviours could revolutionize efforts to mitigate the impact of SLF and other planthoppers. Thus providing alternative management strategies that would decrease insecticide use in fighting these pests.

**Figure 1.**
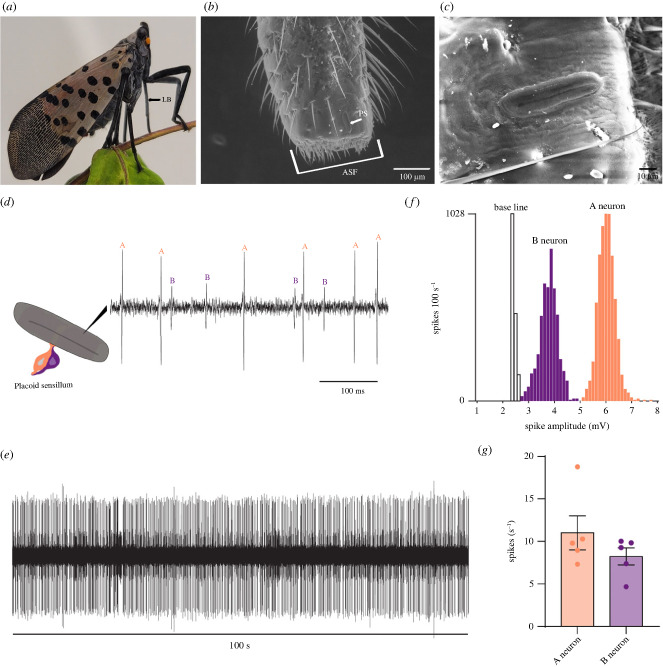
The subapical labial sensory organ in SLF. (*a*) Orientation of mouthparts during feeding in a SLF female. LB, labium. (*b*) Scanning electron microscopy image of the distal part of the fifth labial segment of a SLF female showing the apical sensory field (ASF) and a placoid sensillum (PS) of the subapical sensory organ. (*c*) Scanning electron microscopy image showing one of the placoid sensilla in a SLF female. The image is rotated 90° relative to (*b*). (*d*) Spontaneous activity of a placoid sensillum in a SLF female. Individual action potentials (spikes) are labelled A or B according to their amplitudes. (*e*) Example trace of a 100 s spontaneous activity from a placoid sensillum in a SLF female. (*f*) The bimodal distribution of spike amplitudes in placoid sensilla. ‘A’ and ‘B’ indicate subpopulations of spikes attributed to neurons A and B. (*g*) Spontaneous activities of the A and B neurons. Error bars are SEM. Replicates were collected from five females.

The destructive mouthparts of SLF (figure 1*a*), like those of other planthoppers, consist of a small cone-shaped labrum, a tubular five-segmented labium, and a stylet fascicle with two inner interlocked maxillary stylets partially surrounded by two shorter mandibular stylets [14,15]. The tip of the labium, which makes direct contact with host plants during feeding, contains specialized sensory structures [15]. These structures are suggested to offer planthoppers essential sensory information influencing their subsequent feeding behaviour [14,15]. These structures include the subapical sensory organ and the apical sensory field (figure 1*b*,*c*). Despite extensive morphological studies, the neuronal and functional organization of these sensory structures remains largely unknown not only in SLF, but, with few exceptions, in all planthoppers [15,16].

In this study, we systematically conducted electrophysiological analysis of the subapical labial sensory organ in SLF, using both moving air stimuli and a battery of 85 ecologically relevant odorants. The analysis revealed in SLF that the paired placoid sensilla of this organ share identical neuronal and functional organization. Additionally, it uncovered that the neuron with the large spike amplitude in this organ can detect both odorants and changes in airflow speed. Notably, this neuron exhibited robust excitatory responses solely to three structurally similar aldehydes and inhibitory responses to 62 other odorants, with 1-nonanol, the only known repellent for SLF, inducing the strongest inhibitory response. By contrast, the co-located neuron with the small spike amplitude exclusively serves as an olfactory detector, exhibiting strong excitatory responses to 17 odorants and inhibitory responses to only three other odorants. Among these 17 excitatory compounds, methyl salicylate, the most known potent attractant for all stages of SLF under laboratory and field conditions, was the best activator for the B neuron. Our study provides the cellular basis for the response of SLF to some of its ecologically relevant stimuli. It also identifies new odorants that hold the potential to mitigate the impact of this invasive planthopper.

## 2. Material and methods

### 2.1. Experimental model

From July to November 2023, SLF adults were collected daily from tree-of-heaven in Milford, CT, and transported to our research laboratories at the Connecticut Agricultural Experiment Station campus in New Haven, CT. Inside the laboratories, they were housed in cages (BugDorm-4M3030 Insect Rearing Cage), and sexes were identified by the diagnostic red valvifers in females [1]. The laboratory was maintained at 23°C and 40% relative humidity. Five new animals were used for each set of experiments.

### 2.2. Scanning electron microscopy images

Scanning electron microscopy images were captured using a Hitachi Tabletop Microscope TM3030Plus from Hitachi High-Technologies Corporation, Tokyo, Japan.

### 2.3. Single-sensillum recordings

A single animal with clipped legs and wings was immobilized in a 1000 µl plastic pipette tip, with its head directed towards the narrower end. The pipette tip was then securely attached to a glass microscope slide, with the animal oriented laterally. The labium was gently fixed with double-sided tape on a cover slide. Subsequently, the slide was placed under a light microscope (BX51WI, Olympus, Tokyo, Japan) equipped with a 50× objective (LMPLFLN 50X, Olympus) and 10× eyepieces.

A reference tungsten electrode (catalogue no. 716000, A-M Systems), electrolytically sharpened to 1 μm tip diameter by dipping it repeatedly in a 10% KNO_3_ solution, was inserted into the animal's abdomen. The recording tungsten electrode, identical to the reference electrode, was inserted gently into the placoid sensilla. Signals were amplified (10×; Syntech Universal AC/DC Probe; http://www.syntech.nl), sampled (10 667 samples s^−1^) and filtered (100–3000 Hz with 50/60 Hz suppression) via a USB-IDAC connection (Syntech) to a computer. Action potentials were extracted using Syntech AutoSpike 32 software.

Responses as the increase (or decrease) in the action potential frequency (spikes s^−1^) were calculated by subtracting the number of action potentials during the 0.5 s preceding the odour stimulation from the number of action potentials during the 0.5 s of odour stimulation.

### 2.4. Continuous airflow

A 20 cm glass tube with an inner diameter of approximately 4 mm supplied activated charcoal-filtered humidified air to the labium. Air, continuous and pulsed, was generated by a Stimulus Air Controller CS-55 V2 from Syntech (https://www.ockenfels-syntech.com/products/stimulus-controllers/). The end of the tube was placed about 1 cm from the preparation, and the continuous airflow near the mounted animal had a speed of approximately 40 cm s^−1^.

### 2.5. Airflow and odorized stimuli

Airflow or odorized stimuli were administered by inserting a Pasteur pipette tip into a hole in the 20 cm tube, which directed the continuous air stream over the labium. The airflow through the pipette was controlled by the Stimulus Air Controller CS-55 V2 from Syntech, providing a 0.5 s pulse. The wind speed at the location of the mounted animal, after the addition of either clean airflow or the odorized stimulus to the continuous airflow, was approximately 50 cm s^−1^, unless another wind speed is specified.

As an odour source, a cellulose filter disc (approx. 1 cm in diameter) was soaked with 50 µl of diluted odorant and placed in a disposable borosilicate glass Pasteur pipette (capacity of 2 ml, Fisher Scientific GSA).

Odorants were purchased from Millipore Sigma in the highest available purity, except for phenethyl isobutyrate, which was acquired from TCI America. These odorants were dissolved in paraffin oil (from Millipore Sigma) at a 10^−2^ dilution unless specific dilutions are indicated.

Odorants were presented one after the other with an interval of at least 60 s between the delivery of each odorant. For dose–response curves, odorants were presented with increasing doses in log steps.

Airflow speed at the location of the mounted animal was measured using a DWYER Anemometer: Hot Wire and Thermistor, 4.5 Digit LCD, with a range from 0 to 30 m s^−1^ and an accuracy of ± 3% (Grainger, item 24A466, model 471B-1).

### 2.6. Statistical analysis

Statistical tests were performed in GraphPad Prism (version 10.1.0). All error bars are SEM. *p < 0.05, **p < 0.01, ***p < 0.001, ****p < 0.0001.

## 3. Results

### 3.1. Subapical labial placoid sensilla in SLF house two neurons

The subapical labial sensory organ in SLF consists of two placoid sensilla located laterally near the tip of the labium (figure 1*a–c*). These sensilla are elliptical, slightly concave and aligned parallel to the longitudinal axis of the labium, enclosed by a double furrow (figure 1*c*). Their surface is irregularly rugulose and multiporous.

To determine the number of neurons housed within these placoid sensilla and to characterize their response profiles, we conducted an extensive series of single sensillum recordings. Our recordings were from approximately 200 placoid sensilla on both sides of the labium in males and females.

We successfully classified the action potentials from these placoid sensilla into two distinct populations, initially based on their distinguishable amplitudes (figure 1*d*). The classification was then confirmed by using the ‘Create Amplitude Histogram’ function of the AutoSpike software and recordings of a 100 s spontaneous firing activity period (figure 1*e*). These histograms once again depicted two populations of spikes (*n* = 5) (figure 1*f*). The simple interpretation of these results is that the two populations of spikes correspond to the activities of two different neurons. Ultrastructural studies in flies and other insects confirmed the equivalence of spike populations to the number of neurons within olfactory sensilla [17–23]. We refer to the neuron with the large amplitude as the ‘A neuron’ and the neuron with the small amplitude as the ‘B neuron’.

The A neuron displayed a spontaneous firing rate of 11 ± 2 spikes s^−1^, and the B neuron exhibited a spontaneous firing rate of 8 ± 1 spikes s^−1^ (figure 1*g*). There were no significant differences in the spontaneous firing rates of these neurons between females and males (electronic supplementary material, figure S1; *p* > 0.05, Mann–Whitney test; *n* = 5).

### 3.2. The A neuron responds to changes in airflow speed

**Figure 2.**
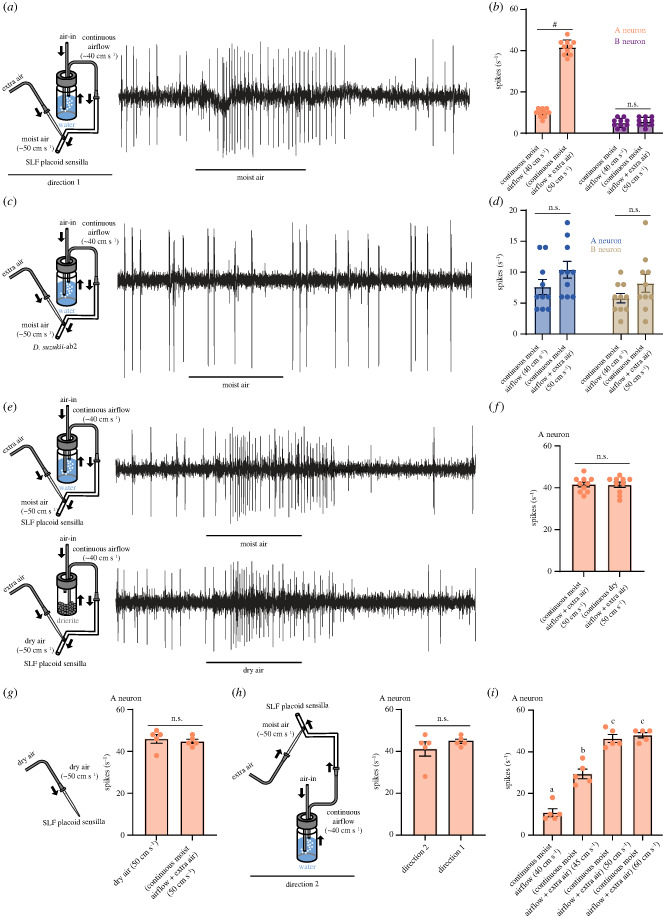
The A neuron of the subapical labial sensory organ in SLF responds to changes in airflow speed. (*a*) Example trace from a placoid sensillum showing the response of the A neuron to the change in airflow speed from approximately 40 cm s^−1^ to approximately 50 cm s^−1^. (*b*) Responses of the A and B neurons to the change in airflow speed from approximately 40 cm s^−1^ to approximately 50 cm s^−1^. ****p < 0.0001; Mann–Whitney test; *n* = 10. Error bars are SEM. Replicates were collected from five females. (*c*) Example trace showing the response of an ab2 sensillum in spotted wing *Drosophila* (*D. suzukii*) to the change in airflow speed from approximately 40 cm s^−1^ to approximately 50 cm s^−1^. (*d*) Responses of the A and B neurons of an ab2 sensillum in spotted wing *Drosophila* (*D. suzukii*) to the change in airflow speed from approximately 40 cm s^−1^ to approximately 50 cm s^−1^. *p* > 0.05; Mann–Whitney test; *n* = 10. Error bars are SEM. Replicates were collected from five females. (*e*) Example traces of the response of the A neuron in SLF to approximately 50 cm s^−1^ airflow speed of continuous moist airflow plus extra air (top) and to approximately 50 cm s^−1^ airflow speed of continuous dry airflow plus extra air (bottom). (*f*) Responses of the A neuron in SLF to approximately 50 cm s^−1^ airflow speed of continuous moist airflow plus extra air and to approximately 50 cm s^−1^ airflow speed of continuous dry airflow plus extra air. *p* > 0.05; Mann–Whitney test; *n* = 10. Error bars are SEM. Replicates were collected from five females. (*g*) Responses of the A neuron in SLF to a change from 0 cm s^−1^ airflow speed of dry air to approximately 50 cm s^−1^ airflow speed of dry airflow and to a change from approximately 40 cm s^−1^ continuous moist airflow to ∼50 cm s^−1^ airflow speed. *p* > 0.05; Mann–Whitney test; *n* = 5. Error bars are SEM. Replicates were collected from five females. (*h*) Responses of the A neuron in SLF to approximately 50 cm s^−1^ airflow speed applied from two different directions. *p* > 0.05; Mann–Whitney test; *n* = 5. Error bars are SEM. Replicates were collected from five females. (*i*) Responses of the A neuron in SLF to changes in airflow speed from ∼40 cm s^−1^ to ∼45 cm s^−1^, ∼50 cm s^−1^, and ∼60 cm s^−1^. One-way ANOVA followed by Tukey's multiple comparison test; *n* = 5. Values indicated with different letters are significantly different. Error bars are SEM. Replicates were collected from five females.

Surprisingly, when we added extra air to the continuous moist airflow, increasing the wind speed from about 40 cm s^−1^ to about 50 cm s^−1^ for 0.5 s, the firing rate went up significantly, from 10 ± 1 to 36 ± 2 spikes s^−1^ at the stimulus onset (*p* < 0.0001, Mann–Whitney test; *n* = 10) (figure 2*a*,*b*). After this, there was a pause of 823 ± 46 ms during which the neuron did not fire before the firing rate returned to the baseline. By contrast, the B neuron remained unaffected by this change in airflow speed (figure 2*a*,*b*; *p* > 0.05, Mann–Whitney test; *n* = 10).

To confirm these findings and eliminate doubts regarding the possibility of artifacts, we applied the same stimulus to one of the three large antennal basiconic sensilla, ab2, found in the fruit pest *Drosophila suzukii*. The identification of ab2 sensillum type in *D. suzukii* was confirmed by using its diagnostic odorants [24,25]. This sensillum type houses two neurons, each with distinct amplitudes and frequencies [24]. As anticipated, neither of these two neurons showed any change in their spontaneous firing rates when airflow speed was increased from 40 cm s^−1^ to 50 cm s^−1^ (figure 2*c*,*d*; *p* > 0.05, Mann–Whitney test; *n* = 10). This outcome supports our conclusion that the response of the A neuron to the increase in airflow speed is indeed specific and not an artifact.

We next tested if the A neuron functions as a humidity sensor by comparing its response to an increased airflow speed, transitioning from 40 cm s^−1^ to 50 cm s^−1^, within continuous moist airflow and continuous dry airflow conditions (figure 2*e*). We found, surprisingly, no difference in the response of the A neuron between dry and moist airflow conditions (figure 2*e*,*f*; *p* > 0.5, Mann–Whitney test; *n* = 10). Furthermore, when dry airflow at a speed of approximately 50 cm s^−1^ was applied directly to the labium, without any mixing with continuous airflow, it produced similar responses to those observed when additional air was introduced to the continuous moist airflow, resulting in a speed of 50 cm s^−1^ (figure 2*g*; *p* > 0.5, Mann–Whitney test; *n* = 5).

We also altered the airflow angle from +45 relative to the midline of the labium (direction 1) to −45 (direction 2). The response of the A neuron to an increased airflow speed (from 40 cm s^−1^ to 50 cm s^−1^) was identical when applied from direction 2 compared to direction 1 (figure 2*h*; *p* > 0.5, Mann–Whitney test; *n* = 5). Furthermore, increasing the airflow speed from approximately 40 cm s^−1^ to 45 cm s^−1^, 50 cm s^−1^, or 60 cm s^−1^ significantly increased the spontaneous firing rate (figure 2*i*; *n* = 5). The maximum response was achieved at an airflow speed of 50 cm s^−1^ and remained constant at 60 cm s^−1^ (figure 2*i*; one-way ANOVA followed by Tukey's multiple comparison test; *n* = 5).

Altogether, these results indicate that the A neuron of the subapical labial sensory organ in SLF is sensitive to changes in airflow speed but not to changes in humidity.

### 3.3. The A neuron exhibits excitatory responses to three aldehydes and inhibitory responses to various odorants

**Figure 3.**
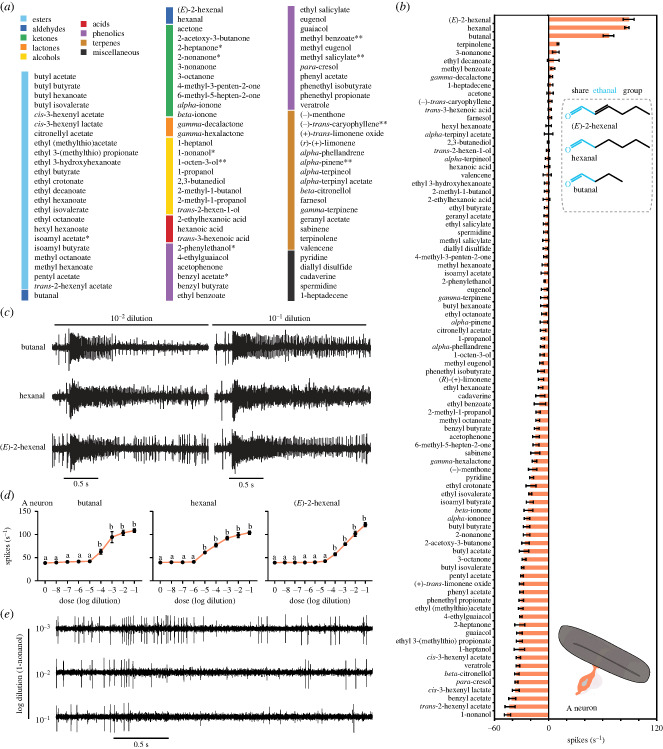
Responses of the A neuron in the subapical labial sensory organ of SLF to odorants. (*a*) The 85 odorants used in the initial screen. *Compounds identified in SLF honeydew. **Compounds identified in SLF host plants. (*b*) Response profile of the A neuron when tested with a 10^−2^ dilution of each odorant. Responses to the diluent control, paraffin oil, were subtracted from each value. Chemical structures of butanal, hexanal, and (*E*)-2-hexanal are indicated. *n* = 5. Error bars are SEM. Replicates were collected from five females. (*c*) Example traces of the response of the A neuron to butanal, hexanal, and (*E*)*-*2-hexenal at 10^−1^ and 10^−2^ dilutions. (*d*) Responses of the A neuron to different dilutions of each of butanal, hexanal, and (*E*)-2-hexenal. One-way ANOVA followed by Dunnett's multiple comparison test; *n* = 5. Values indicated with different letters are significantly different from the control. Error bars are SEM. Replicates for each odorant were collected from five females. (*e*) Example traces of the response of the A neuron to 1-nonanol at 10^−1^, 10^−2^, and 10^−3^ dilutions.

To determine if the two neurons within the placoid sensilla of SLF respond to odorants and comprehensively characterize their odour response profiles, we screened them with a battery of 85 odorants (figure 3*a*). This battery covered a wide range of chemical classes, including acids, alcohols, aldehydes, esters, ketones, lactones, phenolics and terpenes. Several of these odorants were previously identified in SLF host plants and honeydew and have been shown to be behaviourally active in laboratory bioassays [6,7,13].

We found that the A neuron exhibited robust excitatory responses exclusively to three aldehydes out of 85 tested odorants (approx. 3.5%): butanal, hexanal, and (*E*)-2-hexenal (figure 3*b*,*c*). The response to each of these three aldehydes was much higher than to the solvent control (paraffin oil) (*p* < 0.01 for butanal, *p* < 0.0001 for each of hexanal and (*E*)-2-hexenal; one-way ANOVA followed by Dunnett's multiple comparisons test; *n* = 5).

These aldehydes are similar in their chemical structures and share an ethanal group in their skeletons (figure 3*b*). Hexanal and (*E*)-2-hexenal possess a distinctive similar smell and are called green leaf volatiles [26,27].

To determine the sensitivity of the A neuron, we examined its response to eight different dilutions (10^−1^–10^−8^) for each of the three aldehydes. Our analysis revealed that all three aldehydes produced similar dose–response curves (figure 3*d*). However, there was a notable difference in detection thresholds. Hexanal had a threshold of 10^−5^ dilution, whereas both butanal and (*E*)-2-hexanal exhibited a threshold of 10^−4^ dilution (figure 3*d*). This indicates that the A neuron is one order of magnitude more sensitive to hexanal than butanal and (*E*)-2-hexenal, positioning hexanal as the primary activator for the A neuron. Notably, this sensitivity pattern was consistent in both males and females, as our analysis showed no statistically significant differences in responses to different dilutions of the three aldehydes between males and females (electronic supplementary material, figure S2; *p* > 0.5, Mann–Whitney test; *n* = 5).

The dose–response experiments also uncovered variations in response dynamics. For instance, hexanal induced responses that persisted beyond the duration of odour stimulus at 10^−1^ and 10^−2^ dilutions (figure 3*c*). Similarly, butanal and (*E*)-2-hexenal exhibited sustained responses, but exclusively at a 10^−1^ dilution (figure 3*c*). This observation indicates that the response dynamic of the A neuron varies across different odorants and concentrations, offering a potential mechanism for distinguishing odour identity and quantity.

The A neuron also displayed inhibitory responses to a wide array of odorants. For 62 out of 85 tested odorants (approx. 73%), the inhibition was 25% or more below the spontaneous firing rate of the A neuron (figure 3*b*,*e*). These odorants were from different chemical groups. Of particular interest, 1-nonanol, an aversive odorant to SLF [7], induced the strongest inhibitory response (figure 3*b*,*e*). This inhibition occurred at only the two highest dilutions tested: 10^−1^ and 10^−2^ (figure 3*e*). The effect of these dilutions persisted long after the stimulation ended (1.56 ± 0.1 s for 10^−1^ dilution and 1.04 ± 0.1 s for 10^−2^dilution) (figure 3*e*), with the duration of inhibition from the 10^−1^ dilution exceeding that of the 10^−2^ dilution (*p* = 0.04, Mann–Whitney test, *n* = 5) (figure 3*e*).

### 3.4. Unlike the A neuron, the B neuron shows excitatory response to many odorants

**Figure 4.**
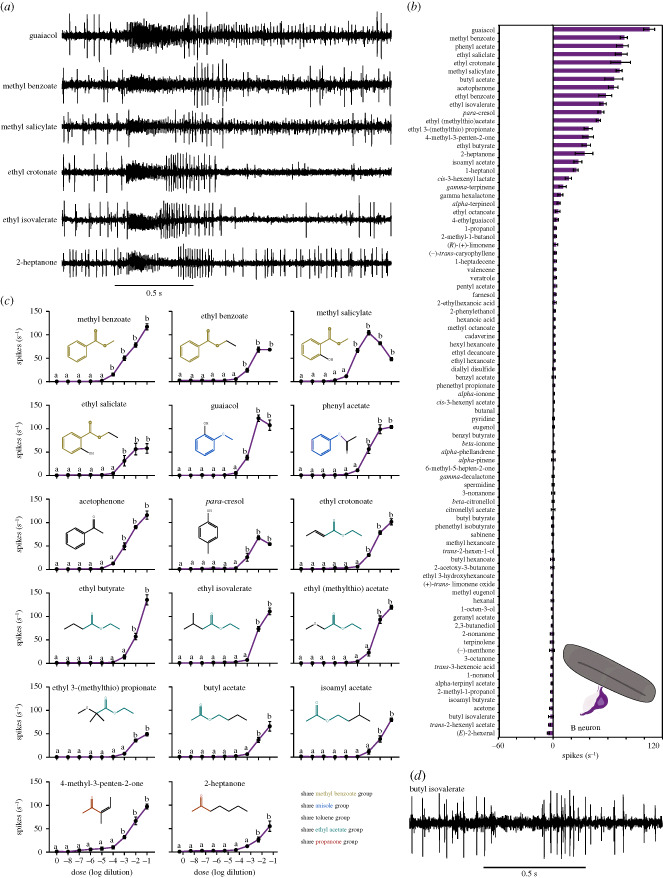
Responses of the B neuron in the subapical labial sensory organ of SLF to odorants. (*a*) Example traces of the response of the B neuron to a 10^−2^ dilution of each of guaiacol, methyl benzoate, methyl salicylate, ethyl crotonate, ethyl isovalerate, and 2-heptanone. (*b*) Response profile of the B neuron when tested with a 10^−2^ dilution of each odorant. Responses to the diluent control, paraffin oil, were subtracted from each value. *n* = 5. Error bars are SEM. Replicates were collected from five females. (*c*) Responses of the B neuron to different dilutions of the best activators. One-way ANOVA followed by Dunnett's multiple comparison test; *n* = 5. Values indicated with different letters are significantly different from the control. Chemical structures are indicated. Error bars are SEM. Replicates for each odorant were collected from five females. (*d*) Example trace of the response of the B neuron to a 10^−2^ dilution of butyl isovalerate. Note that butyl isovalerate inhibited both the A and B neurons.

Unlike the A neuron, the B neuron displayed strong excitatory responses of more than 25 spikes s^−1^ to 17 out of the 85 tested odorants (approx. 20%) (figure 4*a*,*b*). The response to each of these 17 odorants was statistically significant compared to the solvent control (*p* < 0.0001 for each odorant; one-way ANOVA followed by Dunnett's multiple comparisons test; *n* = 5).

These 17 excitatory odorants include eight phenolic compounds, seven aliphatic esters, and two aliphatic ketones (figure 4*c*). Within the eight phenolic compounds, four (methyl benzoate, ethyl benzoate, methyl salicylate, and ethyl salicylate) incorporate methyl benzoate into their structures, two (guaiacol and phenyl acetate) carry anisole, and two (acetophenone and *para*-cresol) contain toluene. The seven aliphatic esters (ethyl crotonate, ethyl butyrate, ethyl isovalerate, ethyl (methylthio) acetate, ethyl 3-(methylthio) propionate, butyl acetate, and isoamyl acetate) all share the common element of ethyl acetate. The two aliphatic ketones (2-heptanone and 4-methy-3-penten-2-one) both feature the propanone group.

At 10^−2^ dilution, 2-heptanone, ethyl crotonate, and ethyl isovalerate produced phasic responses that peaked and decayed quickly to baseline or to a level near baseline before the end of the stimulus duration (figure 4*a*). By contrast, guaiacol, methyl benzoate, and methyl salicylate elicited tonic responses that persisted for a considerable time after the end of the stimulus duration (figure 4*a*).

The detection thresholds for the eight phenolic compounds fell within the range of 10^−3^ to 10^−4^ dilutions, while the nine aliphatic compounds (seven aliphatic esters and two aliphatic ketones) showed detection thresholds between 10^−2^ and 10^−3^ dilutions (figure 4*c*). Interestingly, only six out of the 17 odorants reached saturation (i.e. the maximum response that declines with increasing concentration), albeit at different dilutions. Specifically, guaiacol, ethyl benzoate, ethyl salicylate, phenyl acetate, and *para*-cresol all achieved saturation at a 10^−2^ dilution, while methyl salicylate reached its saturation point at a 10^−3^ dilution, establishing methyl salicylate as the best activator for the B neuron (figure 4*c*). These findings indicate that the B neuron responds to a diverse range of odorants with varying sensitivities and saturation levels. Additionally, we found that the responses of both males and females to various dilutions of ethyl benzoate and ethyl crotonate were statistically similar (electronic supplementary material, figure S3; *p* > 0.5, Mann–Whitney test; *n* = 5).

Moreover, we found that, in contrast to the A neuron, which was inhibited by an array of odorants, the B neuron exhibited inhibitory responses to only three odorants: butyl isovalerate, *trans*-2-hexenyl acetate, and (*E*)-2-hexenal (figure 4*b*,*d*). The inhibition caused by each of these three odorants was 25% or more below the spontaneous firing rate of the B neuron.

## 4. Discussion

We have characterized the neuronal and the functional organization of the subapical labial sensory organ in SLF. This sensory structure is not only present in SLF but also conserved across at least two other planthopper families, Dictyopharidae and Ricaniidae, highlighting its significance in facilitating the interaction of planthoppers with their host plants. Our findings reveal that this organ comprises two identical placoid sensilla, each housing two different neurons. The A neuron, characterized by a large amplitude, is a dual-modal neuron, responding to changes in airflow speed and to odorants. By contrast, the B neuron, with a small amplitude, exclusively responds to odorants.

### 4.1. Coding of changes in airflow speed at the periphery

We have found, unexpectedly, that the A neuron of the subapical labial sensory organ in SLF responds to changes in airflow speed, while the B neuron remains unaffected. This response was consistent across all trials and was specific to the A neuron. Importantly, this response was independent of humidity.

Previous studies have not proposed a role for the subapical labial sensory organ in detecting changes in airflow speed in SLF or any other planthoppers [7,8]. Part of the reason for this is the multiporous nature of the paired placoid sensilla of this organ, typically linked with an olfactory function. Additionally, other insects have specialized mechanosensory organs, distinct from the two placoid sensilla of the subapical labial sensory in SLF, to detect changes in air movement [28–31].

However, the specific positioning and size of the subapical labial sensory organ in SLF render them ideally equipped for this function. The paired placoid sensilla of this organ are uniquely oriented perpendicular to the food surface during feeding (figure 1*a*), a strategic placement that ensures their continuous exposure to environmental cues while the insect is engaged in the act of feeding. This orientation positions them adeptly to detect fluctuations in air speed. Furthermore, each of the two placoid sensilla of this organ possesses a notably generous surface area, measuring approximately 52 µm in length and 21 µm in width (figure 1*b*,*c*) [15].

This suggests that the A neuron of the subapical labial sensory organ in SLF may function as a flux detector for sudden changes in air pressure or mechanical forces caused by air movement, rather than the overall air pressure or force of the surrounding air at any given moment. This parallels the behaviour of neurons specialized for thermosensation (sensing hot or cold) in insects, which respond to changes in temperature rather than the absolute temperature values [32,33].

Intriguing, but less understood is how an olfactory neuron within a multiporous sensillum detects changes in airflow speed. This detection could be attributed simply to the expression of a mechanoreceptor on the olfactory dendrite. Notably, a precedent for a similar sensory modality specifically for changes in humidity levels has been reported in *Drosophila melanogaster*. In *D. melanogaster*, olfactory receptor *Or42b*-expressing neurons within the multiporous antennal basiconic sensillum type 1 (ab1) are known detectors for food-related scents [19,34,35]. These olfactory neurons were recently shown to also participate in hygrosensation through the expression of the mechanosensitive molecule *TMEM63* within them [36]. Interestingly, the sensilla that house the dendritic branches of *Or42b* neurons exhibit a shape change in response to increasing humidity [36]. This observation suggests that humidity stimuli can be converted into mechanical force on the dendritic membrane.

Given the absence of known receptor genes for detecting wind or changes in airflow speed in insects, including the model organism *D. melanogaster*, it is intriguing to explore whether SLF has developed a unique receptor gene for this purpose. Another fascinating avenue for investigation involves examining whether the olfactory receptors in SLF possess dual functionality, enabling them to detect both odorants and changes in airflow speed.

### 4.2. Odour coding by the neurons of the subapical labial sensor organ in the spotted lanternfly

Both the A and B neurons of the subapical labial sensory organ in SLF respond to odorants. The A neuron exhibited robust excitatory responses exclusively to three structurally similar aldehydes: butanal, hexanal, and (*E*)-2-hexenal. Notably, these aldehydes have not been identified in previous studies that aimed to discover physiologically and behaviourally active compounds for SLF [6,7,13]. This oversight can be attributed, in part, to the emphasis of prior investigations on the antenna, another olfactory organ in SLF [37]. Additionally, the volatility of butanal during gas chromatography runs contributes to its elusive nature, as it tends to emerge from the instrument within the solvent peak. Hexanal and (*E*)-2-hexenal fall under the category of green leaf volatiles, which are responsible for the smell of freshly cut grass [26,27,38,39]. Green leaf volatiles are known to provide plants with both direct protection by inhibiting or repelling herbivores and indirect protection by attracting predators of the herbivores themselves [26,27,38,39]. Consequently, it remains a future endeavour to measure the behavioural response of SLF to these three aldehydes and potentially use them to develop new strategies for managing the invasion of this species.

The specificity of excitatory responses in the A neuron of the subapical labial sensory organ in SLF to only three aldehydes resembles that observed in neurons detecting cues of particular biological importance in *D. melanogaster*, such as ab4B [40]. This neuron exclusively detects geosmin, a compound associated with the presence of harmful microbes that pose a threat to flies. Activation of this neuron serves as an indicator of potential danger. Similarly, in *D. melanogaster*, the ab10B neuron exclusively detects iridomyrmecin, actinidine, and nepetalactol, which are the pheromones of flies' natural enemies [41]. Activation of the ab10B neuron in response to these pheromones is sufficient to induce aversion in flies. In mosquitoes and moths such specific pathways have also been well described [20,42–49]. The parallels drawn from these findings shed light on the specialized and evolutionarily conserved roles of specific olfactory neurons in responding to ecologically relevant cues.

In contrast to the A neuron, the B neuron appears to function as a general detector. This neuron exhibited strong excitatory responses to a broader spectrum of 17 odorants. This spectrum includes eight phenolic compounds, seven aliphatic esters, and two aliphatic ketones. Notably, several of these odorants, such as methyl salicylate, methyl benzoate, and 2-heptanone, have been found to be attractive to SLF in behavioural bioassays [6,7,13]. Of particular interest, methyl salicylate, identified as the most potent activator of the B neuron in our study, is a major volatile associated with the preferred host plant for SLF, *Ailanthus altissima* (also known as the tree-of-heaven) [6]. Moreover, methyl salicylate is attractive to adults and nymphs of SLF under laboratory conditions and, to some extent, under field conditions as well [6,50].

The two neurons of the subapical labial sensory organ in SLF exhibited distinct excitatory odour response profiles, even at the highest tested dilution (10^−2^) (figures 3*b* and 4*b*). However, it is worth noting that our examination has been limited to a single olfactory organ, the subapical labial sensory organ. Future exploration of other organs and sensory fields in SLF would be essential to comprehensively reevaluate this finding. Previous evidence from work on the behaviour of *D. melanogaster* larva indicates that having several receptors to the same odorant but with different detection thresholds is critical to successfully discriminate specific plant odorant mixtures [51,52]. In this regard, it would be interesting to know if SLF discriminates host from non-host plants through specific combinations of odorants that activate separate neurons or specific combinations of odorants that activate many neurons over a large range of concentrations.

Another interesting finding regarding neurons in the subapical labial sensory organ of SLF is the inhibition of the A neuron by a substantial number of odorants (62 out of 85 tested odorants). Among these odorants, 1-nonanol induced the strongest inhibitory response. Notably, 1-nonanol was shown to repel SLF in laboratory behavioural bioassays [7]. This observation aligns with parallel research, as inhibition in sensory neurons has been previously correlated with repellency in *Drosophila* larvae [52].

## 5. Conclusion

In summary, our work characterizes at the cellular level the first labial sensory organ to be investigated in SLF. This exploration uncovers an innovative peripheral coding mechanism, where a single neuron responds to both odorants and changes in airflow. It also establishes foundational knowledge for understanding the behavioural response of SLF to ecologically relevant stimuli. Furthermore, our work identifies novel compounds that have the potential to revolutionize our approach aimed at mitigating the devastating effects of this invasive species.

## Data Availability

The data supporting the results in this paper are available on Dryad [53]. Supplementary material is available online [54].
